# The Chromatin Response to Double-Strand DNA Breaks and Their Repair

**DOI:** 10.3390/cells9081853

**Published:** 2020-08-07

**Authors:** Radoslav Aleksandrov, Rossitsa Hristova, Stoyno Stoynov, Anastas Gospodinov

**Affiliations:** Roumen Tsanev Institute of Molecular Biology, Bulgarian Academy of Sciences, Acad. G. Bonchev Str. 21, 1113 Sofia, Bulgaria; raleksandrov@bio21.bas.bg (R.A.); hristova_r@bio21.bas.bg (R.H.); stoynov@bio21.bas.bg (S.S.)

**Keywords:** DNA damage response, double-strand DNA break repair, non-homologous end joining, homologous recombination, chromatin dynamics in DNA repair, synthetic lethality, PARP inhibitors, cancer, anticancer drug therapies

## Abstract

Cellular DNA is constantly being damaged by numerous internal and external mutagenic factors. Probably the most severe type of insults DNA could suffer are the double-strand DNA breaks (DSBs). They sever both DNA strands and compromise genomic stability, causing deleterious chromosomal aberrations that are implicated in numerous maladies, including cancer. Not surprisingly, cells have evolved several DSB repair pathways encompassing hundreds of different DNA repair proteins to cope with this challenge. In eukaryotic cells, DSB repair is fulfilled in the immensely complex environment of the chromatin. The chromatin is not just a passive background that accommodates the multitude of DNA repair proteins, but it is a highly dynamic and active participant in the repair process. Chromatin alterations, such as changing patterns of histone modifications shaped by numerous histone-modifying enzymes and chromatin remodeling, are pivotal for proficient DSB repair. Dynamic chromatin changes ensure accessibility to the damaged region, recruit DNA repair proteins, and regulate their association and activity, contributing to DSB repair pathway choice and coordination. Given the paramount importance of DSB repair in tumorigenesis and cancer progression, DSB repair has turned into an attractive target for the development of novel anticancer therapies, some of which have already entered the clinic.

## 1. Introduction

DNA carries all hereditary genetic instructions dictating and regulating cellular functions and fate. Following the discovery of its notable double helical structure, DNA was considered a fairly stable molecule. However, it did not take much time before scientists discovered that DNA is subject to the damaging effects of multiple mutagens that generate a remarkable diversity of harmful lesions [1]. On the one hand, the emergence of mutations in DNA, which generate new alleles or change the position or the number of the genes in the genome, is the driving force of evolution since these mutations are the “raw material” upon which evolution exerts selection [2]. However, on the other hand, most mutations are deleterious and disrupt the function(s) of the damaged genes, leading to various pathologies. Accumulation of DNA lesions has been implicated in cell cycle arrest, cell senescence and death, aging, tumorigenesis, diverse developmental defects, and neurodegenerative diseases [3,4]. It has been estimated that every nucleated cell in the human body suffers approximately 70,000 DNA lesions every day, and external sources of mutation only increase this number. Taking into account the huge number of mutations arising constantly and their potential to inflict detrimental effects, it is logical that for billions of years of evolution, cells have been developing mechanisms for the detection and repair of DNA lesions [2,3,5].

Research in many laboratories over the years revealed an intricate network of hundreds of proteins and protein complexes organized in discrete pathways that recognize and repair specific types of DNA lesions [6]. These DNA repair pathways are precisely coordinated with the progression of the cell cycle and function in the immensely complicated and dynamic context of the chromatin [7,8,9]. The importance of the DNA damage response (DDR) is demonstrated by the fact that almost all types of cancer exhibit some kind of DNA repair deficiency [10] and mutations in genes coding for essential DNA repair factors are often associated with considerably increased predisposition to cancer and/or premature aging [3]. 

In this review, we discuss the mechanisms that eukaryotic cells employ to repair double-strand DNA breaks (DSBs)—the most toxic type of DNA lesions. Especially, we focus on the elaborate network of dynamic chromatin modifications and extensive chromatin remodeling, which facilitate and regulate DSB repair pathway choice and implementation. Being of paramount importance to the process of tumorigenesis, DSB repair mechanisms and DSB repair-associated chromatin changes have turned into an attractive target for the development of novel therapeutic strategies for treating cancer patients [11,12]. We consider the numerous tactics that have emerged to target DSB repair pathways with an emphasis on the therapeutic paradigm of synthetic lethality. 

## 2. Tying up Loose Ends—Overview of DNA Double-Strand Break Repair Pathways

### 2.1. Sources of Damage

Mutagens can broadly be divided into endogenous and exogenous by their source of origin. Endogenous mutagens are generated inside the cells of the organism and are generally chemical in nature comprising Reactive Oxygen Species (ROS) generated in the oxidative reactions of the metabolism and other highly reactive byproducts of certain enzymatic reactions such as formaldehyde formed during histone demethylation [13,14]. Exogenous mutagens include a large cohort of DNA-damaging compounds and high energy electromagnetic radiations such as UV-, X-ray, and gamma-radiation that are able to damage DNA either directly or indirectly [3].

It has been shown that ROS generated during oxidative metabolism are among the primary sources of double-strand DNA breaks in eukaryotic cells. Oxygen-containing radicals such as hydroxyl radicals (●OH) and superoxide anion radicals (●O_2_^−^) are extremely reactive particles capable of damaging molecules in their immediate vicinity, including DNA, creating a significant DSB burden if their production is left unattended [13,15,16]. In addition, tumorigenesis requires the activation of oncogenes, which in turn stimulate the uncontrolled proliferation of the cells. To achieve this, oncogenes increase DNA replication origin firing leading to replication stress due to altered nucleotide levels and replication fork speed. Replication stress may cause replication fork stalling and collapse, generating copious amounts of DSBs during the S phase of the cell cycle [17,18]. Moreover, increased replication rates could lead to transcription-replication conflicts resulting in DNA breaks that fuel genomic instability [19]. Both DNA and RNA polymerases lead to the generation of superhelical strain in DNA during transactions such as DNA replication, repair, and transcription. This tension is relieved by the action of a specific group of enzymes dubbed topoisomerases, which induce the formation of either single- or double-strand breaks in DNA to relax the strained DNA molecules [20]. Lesions that are present on DNA molecules during DNA-based processes increase the probability for abortive topoisomerase reactions that could lead to the formation of DSBs [2,20,21]. Certain cellular processes include the controlled formation of DNA breaks. For example, V(D)J and immunoglobulin heavy chain class switch recombination entail the formation of DSBs in order for B- and T-lymphocytes to form their extensive repertoire of antibodies or cell surface antigen receptors [22]. Meiotic crossing over, one of the main processes creating genetic diversity during sexual reproduction, also involves the regulated induction of DNA breaks in order for homologous chromosomes to exchange genetic material in gametogenesis [23].

Environmental factors such as ionizing radiation and certain chemicals can induce the formation of excessive numbers of DSBs. High energy X-ray and gamma-radiation can lead to DSBs either directly or indirectly through the generation of ROS. Low doses of radiation received during procedures such as medical imaging or even air travel can inflict damage and increase the basal levels of DSBs [3]. Mutagenic chemical compounds contained in cigarette smoke (benzo[a]pyrene) or biological toxins such as the aflatoxins that are present in the spores of *Aspergillus* fungi covalently bind to DNA and induce the formation of DSBs following replication fork stalling and collapse [24,25]. Acetaldehyde produced during ethanol oxidation can induce inter-strand DNA crosslinks (IPCs) and DNA-protein crosslinks (DPCs) that may lead to DSB formation during DNA repair or transcription [14,26].

Having in mind the complexity of DSB induction and the consequences organisms suffer from untimely and mutagenic DSBs, cells must have evolved specific molecular mechanisms to cope with the genomic instability posed by double-strand DNA breaks.

### 2.2. Pathways for Double-Strand DNA Breaks Repair

**Figure 1:**
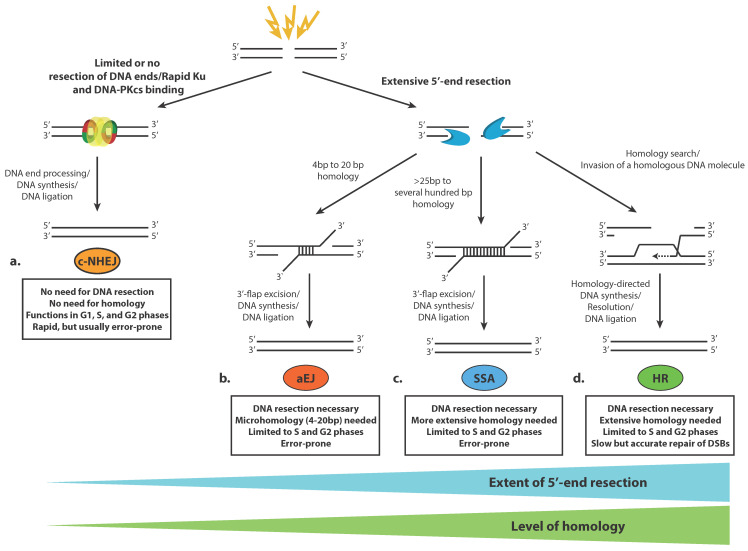
General overview of double-strand DNA break repair pathways in eukaryotic cells. (**a**) Classical non-homologous end joining (c-NHEJ), (**b**) Alternative end joining (aEJ), (**c**) Single-strand annealing (SSA), (**d**) Homologous recombination (HR).

Four different DSB repair pathways have been described thus far (Figure 1). Although these pathways are very specific in their mechanisms and outcomes, they share many common proteins and are substantially interdependent [18]. The two main DSB repair pathways are the classical non-homologous end joining (c-NHEJ) pathway and homologous recombination (HR). c-NHEJ proteins are rapidly assembled on the ends of broken DNA molecules, and following limited or no processing of the DSBs, those ends are ligated (Figure 1a). c-NHEJ does not require broken ends to contain homology, although the presence of microhomology of up to 4bp can speed up the process and increase its accuracy [27]. c-NHEJ entailing end processing of the breaks is typically error-prone and may lead to chromosome rearrangements such as deletions and translocations, especially if many DSBs are formed in close proximity. HR, on the other hand, is a usually error-free DSB repair mechanism that requires extensive end processing of the DNA breaks (5′-end resection) in order to use the long ssDNA regions that have been generated to invade a homologous DNA sequence that is used as a template to achieve accurate repair of the DSB (Figure 1d). The choice between these two major pathways for DSB repair is linked to the progression of the cell cycle. Since c-NHEJ does not require homology, it is active throughout all phases of the cell cycle. The need for extensive homology for HR restricts this mechanism to the S and G2 phases of the cell cycle when following DNA replication, an identical DNA sequence that can serve as a template for error-free DSB repair is present in the nucleus [28]. The complicated cell cycle-dependent sequence of events, which leads to DSB repair pathway choice, entails extensive modifications and remodeling of the chromatin in the vicinity of the break that are thoroughly discussed below.

In addition, two alternative, less well-understood pathways for DSB repair have been defined. Alternative end joining (aEJ) may take place between ssDNA ends that have been generated following 5′-end resection (Figure 1b). Resection may reveal microhomologous regions (usually in the range 4 to 20 bp) between the processed DNA ends, which can bind to each other, and after removal of 3′-DNA flaps, the ends are ligated. Single-strand annealing (SSA) involves the annealing of more extensively processed DNA ends that carry wider homologous sequences, usually several tens to over a hundred nucleotides long (Figure 1c). Such homologous sequences are typically present if tandem repeats flank the DSB site, which, after resection, can anneal, and following DNA flap excision and limited DNA synthesis, can be ligated to restore the integrity of the double-stranded DNA molecule. SSA and aEJ are inherently mutagenic types of DSB repair since ligation of processed ends is preceded by 3′-flap excision, which generates deletions in DNA. Both SSA and aEJ demand DNA resection of the broken ends, meaning that they are active, like HR, in the S and G2 phases of the cell cycle. There is still a debate if SSA and aEJ are bona fide DSB repair mechanisms or serve as backup mechanisms when the two major pathways, c-NHEJ and HR, are compromised in some way [18,27].

Notably, many widely-used anticancer drugs, such as platinum salts, DNA alkylating agents, and topoisomerase inhibitors, are DNA-damaging agents that suppress cancer cell proliferation by inducing various types of DNA lesions, including DSBs [29], that lead to cell cycle arrest and cell death. Therefore, the proficient repair of DSBs may grant cancer cells with resistance towards these compounds and render chemotherapy largely inefficient [30,31,32]. Enhancing our knowledge of the mechanisms that simultaneously govern DSB repair and regulate the chromatin context in the course of the repair process is pivotal in designing more rational and beneficial chemotherapy regimens for cancer patients.

### 2.3. Classical Non-Homologous End Joining (c-NHEJ)

**Figure 2:**
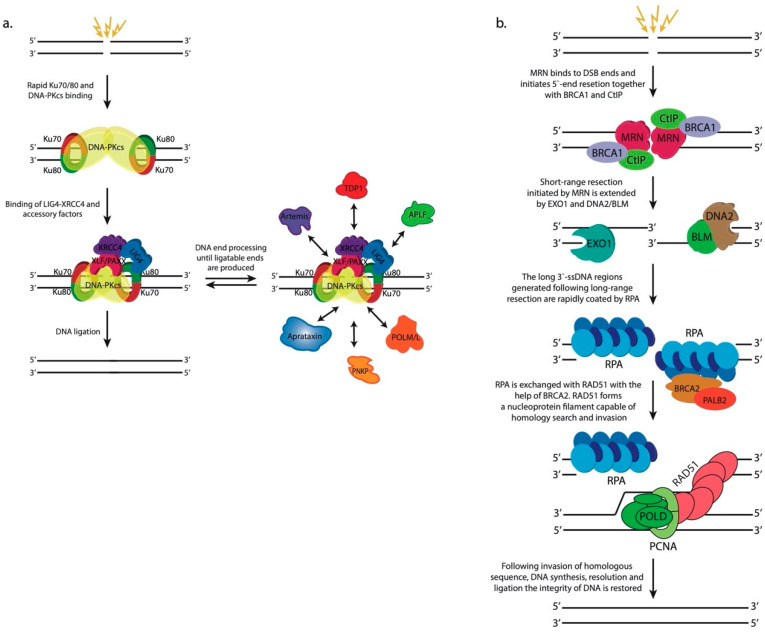
Schematic representation of the major double-strand DNA breaks (DSB) repair pathways. (**a**). Classical non-homologous end joining (c-NHEJ). The rapid binding of Ku and DNA-PKcs creates a long-range synapse that stabilizes and protects DNA ends. Binding of LIG4-XRCC4 and accessory factors induce rearrangements leading to the formation of a short-range synapse that is competent for end processing. DNA ends that are not ligatable may be subjected to several cycles of end processing until ligatable ends are generated. (**b**) Homologous recombination. The MRN complex binds to DSB ends and associates with CtIP and BRCA1 to catalyze short-range 5′-end resection. EXO1 and DNA/BLM2 extend the resected region to several hundred or more than a thousand nucleotides away from the break site. The long 3′-ssDNA tails are rapidly bound by the heterotrimeric RPA complex, which is displaced by BRCA2-PALB2, which is necessary for the formation of a RAD51-ssDNA nucleofilament that is capable of invading a homologous DNA sequence. DNA synthesis performed by DNA polymerases in cooperation with the processivity factor PCNA restores the damaged region, and following resolution and ligation, the integrity of DNA is restored perfectly.

c-NHEJ is initiated by the binding of the heterodimer Ku70-Ku80 complex to the broken ends of DNA molecules (Figure 2a). The Ku complex is highly abundant in eukaryotic cells and exhibits high affinity towards blunt DNA ends or ends with short single-strand overhangs [33,34]. The Ku heterodimers bound to both ends of the break act as a platform for the further binding of DNA-dependent protein kinase catalytic subunit (DNA-PKcs), forming the DNA-PK complex [35]. DNA-PKcs is a very large protein belonging to the family of phosphoinositide 3-kinase (PI3K)-related kinases (PIKKs) [36]. DNA-PKcs autophosphorylates itself and, in addition, phosphorylates the chromatin in the vicinity of DSBs and many downstream c-NHEJ factors contributing to their timely recruitment and activation. Blunt DNA ends or ends bearing short homologous overhangs are efficiently and rapidly sealed by the action of the DNA ligase 4–X-ray repair cross-complementing protein 4 (LIG4-XRCC4) complex. XRCC4 is an essential binding partner of LIG4 and stimulates its enzymatic activity [37]. Two more proteins, XRCC4-like factor (XLF, also known as Cernunnos) and Paralogue of XRCC4 and XLF (PAXX), interact with the LIG4-XRCC4 complex and exhibit scaffolding functions facilitating the proper positioning of DNA ends prior to ligation [38,39]. However, broken DNA ends are often not complementary and/or contain modified nucleotides, which necessitates their processing prior to ligation, often in a cyclic fashion. The array of processing enzymes that is used on a particular occasion depends on the nature of the modifications carried by the damaged DNA ends. Artemis is a nuclease recruited to DSBs through its interaction with DNA-PKcs [40]. This interaction stimulates Artemis, which exhibits endonuclease activity and acts upon short overhangs or hairpins. Another c-NHEJ nuclease is aprataxin and PNKP-like factor (APLF), which displays both apurinic-apyrimidinic (AP) endonuclease activity and 3′-5′ exonuclease activity [41,42]. Polynucleotide kinase 3′-phosphatase (PNKP) is a bispecific enzyme, which adds phosphate groups to 5′-ends of DSBs that are lacking one and remove 3′-phosphates which hinder the ligation step [43]. Tyrosyl DNA phosphodiesterase 1 (TDP1) eliminates 3′-phopshoglycolates (3′-PGs) [44] and aprataxin [45] removes adenosine monophosphate (AMP) residues attached to the 5′-ends of DNA breaks owing to abortive DNA ligation reactions. In addition to nucleases and end-processing enzymes, two DNA polymerases belonging to the X-family of DNA polymerases, DNA polymerase λ and DNA polymerase μ, are employed by c-NHEJ. Both polymerases are able to add nucleotides in a template-dependent or template-independent fashion to the 3′-ends of the break until ligatable ends are achieved [34,46]. Typically, as a result of end processing by the abovementioned enzymes, the ends of the breaks drop or gain several nucleotides creating microdeletions or microinsertions, respectively [27]. Therefore, c-NHEJ is considered inherently mutagenic pathway for DSB repair.

### 2.4. Homologous Recombination (HR)

The hallmark of homologous recombination compared to c-NHEJ is the extensive 5′-end resection of broken DNA ends that precedes the homology-directed repair of the break (Figure 2b). This processing step is implemented in a highly coordinated and regulated fashion by several nucleases and helicases [47]. HR is initiated by the binding of the heterotrimeric MRE11-RAD50-NBS1 (MRN) complex to DSBs. MRN is a highly dynamic protein complex that interacts with multiple downstream HR factors [48]. Its binding to the CtBP-interacting protein (CtIP, also known as RBBP8) activates the endonuclease activity of the MRE11 subunit, which nicks the strand that ends with a 5′-terminus at the DSB. This nick may be introduced up to several hundred bp away from the break site. MRE11 also exhibits 3′-5′ exonuclease activity, which is responsible for the short-range resection of the nicked strand from the nick as far as to the break site creating a long 3′-ssDNA tail [49,50,51,52].

The MRN complex recruits the prominent DNA damage response (DDR) kinase ataxia-telangiectasia mutated (ATM) through direct interaction between ATM and MRN’s NBS1 subunit [53]. ATM, like DNA-PKcs, is a member of the PIKK family of protein kinases, and following its recruitment to DSB sites, ATM phosphorylates Ser139 of H2A.X (termed γH2A.X after S139 phosphorylation) [54] in the vicinity of the break initiating a cascade of chromatin modifications discussed in detail later in this review. Briefly, γH2A.X serves as a binding site for Mediator of DNA damage checkpoint protein 1 (MDC1), which in turn is modified by ATM [55,56]. Phosphorylated MDC1 recruits the E3 ubiquitin ligase Ring Finger Protein 8 (RNF8) that launches a highly complex ubiquitination cascade of the chromatin [57]. RNF8 polyubiquitinates histone H1 that is bound by Ring Finger Protein 168 (RNF168) [58]. RNF168 ubiquitinates histones H2A/H2A.X at lysine residues K13 and/or K15, which are bound by downstream HR factors [59,60]. The ubiquitination cascade of the chromatin eventually recruits the heterodimeric Breast cancer type 1 susceptibility protein-BRCA1 Associated RING Domain 1 (BRCA1-BARD1) protein complex [61,62,63]. BRCA1-BARD1 is the most important mediator of 5′-end resection that channels DSB repair towards HR. BRCA1-BARD1 interacts with CtIP and MRN in a cell cycle-dependent manner to stimulate MRE11′s activity and initiate resection [64]. The short-range resection completed by the MRN complex is further extended (long-range resection) by exonuclease 1 (EXO1) and the heterodimeric DNA2/Bloom syndrome protein (DNA2/BLM) nuclease-helicase complex [65,66,67,68]. Long-range 5′-end resection generates extensive 3′-ssDNA (at least several hundred bp) that is readily coated by the heterotrimeric replication protein A (RPA) complex [69]. RPA affinity for ssDNA is significant and it must be evicted for the final steps of HR to commence. BRCA1-BARD1 interacts with Partner and localizer of BRCA2 (PALB2) that recruits the Breast cancer type 2 susceptibility protein (BRCA2) to the long 3′-ssDNA regions. BRCA2 is essential for promoting RPA exchange with RAD51 along the single-strand DNA regions [70,71]. RAD51 binds to ssDNA forming helical RAD51-ssDNA nucleoprotein filaments that are capable of homology search and invasion of a homologous DNA sequence [72,73]. BRCA1-BARD1 facilitates homologous pairing and if sufficient base pairing occurs between the invading RAD51-coated strand and the invaded DNA molecule the non-base-paired strand of the invaded molecule is displaced in the form of a loop (D-loop) [74] and the 3′-end of the invasion strand is engaged by DNA polymerase δ or translesion DNA polymerases [75]. DNA polymerases extend the 3′-end of the invasion strand past the break using the invaded homologous strand as a template. This repair DNA synthesis is followed by resolution, annealing, and ligation of the extended invasion strand to the other end of the DSB on the original DNA molecule, effectively resealing the damaged region.

### 2.5. Alternative End Joining (aEJ)

Contrary to c-NHEJ, alternative end joining requires 5′-DNA end resection of the DSB ends, which usually ranges between 15 and 100 nucleotides from the site of the breaks. This resection step is essential as it exposes short (between 2 bp and 20 bp) microhomology sequences that can anneal and serve as the basis for further rejoining of the break [27]. The resection is accomplished, as in HR initiation, by the MRN complex and CtIP [76]. It has been shown that poly(ADP-ribose) polymerase 1 (PARP1), an abundant DNA damage sensor, promotes aEJ possibly through competition with the Ku heterodimer for DSB binding [77,78]. PARP1 binding to DNA activates its enzymatic activity, and PARP1 starts to form long negatively-charged poly(ADP-ribose) (PAR) chains on itself and on the chromatin proteins surrounding the break (PARylation) that serve as a platform for the recruitment of downstream DNA repair factors [79]. PARP1 is necessary for the recruitment of DNA polymerase Θ, which is the central mediator of aEJ [80]. Pol Θ binds to the 3′-ends of short annealed microhomology sequences stabilizing their association and extends the 3′-ends of the DNA strands to strengthen the association between the ends of the break [81]. If the 3′-ssDNA regions have annealed upstream and their ends are protruding, the association of Pol Θ is preceded by the action of several nucleases such as the xeroderma pigmentosum group F-Excision Repair Cross-Complementation Group 1 (XPF-ERCC1) nuclease, Artemis, and APLF, which remove the 3′-flaps setting the stage for Pol Θ binding [27]. Following Pol Θ-mediated fill-in synthesis at both sides of the annealed region, the break is sealed either by DNA ligase 1 or by the DNA ligase 3-X-ray repair cross-complementing protein 1 (LIG3-XRCC1) complex [82]. aEJ is inherently mutagenic DSB repair pathway since it leads to deletions due to 3′-flaps removal prior to fill-in synthesis and ligation. Currently, it is unclear if aEJ is a bona fide DSB repair mechanism and processes a subset of DSBs that cannot be engaged by c-NHEJ or acts as a backup mechanism when cNHEJ is compromised [18]. The importance of aEJ is apparent in HR-deficient cells, which are heavily dependent on Pol Θ-mediated aEJ for their survival, and this dependency may be exploited in the clinic, but further research is necessary to assess aEJ contribution to DSB repair in normal cells [83,84].

### 2.6. Single-Strand Annealing (SSA)

Single-strand annealing, compared to aEJ, also requires 5′-end resection of DSB ends, but the extent of the resection is larger since SSA may be accomplished between homologous sequences located along the 3′-ssDNA tails that are significantly longer—in the range between 25 to several hundred nucleotides [85]. Such homologous sequences are most often available due to the presence of tandem repeats flanking both ends of the break [18]. The long-range resection necessary for SSA is initiated by MRN and CtIP and extended by EXO1 and DNA2/BLM [86]. ssDNA is coated by RPA, but the final steps of SSA are RAD51-independent as opposed to HR. The annealing between the complementary ssDNA regions is facilitated by the RAD52 protein, which displaces the RPA molecules coating the ssDNA. If 3′-flaps are present, they are excised by the XPF-ERCC1 endonuclease rendering SSA obligatory mutagenic DSB repair pathway since the intervening sequences between the complementary regions are lost [18,27,87,88].

## 3. Chromatin Response to Double-Strand DNA Breaks

**Figure 3:**
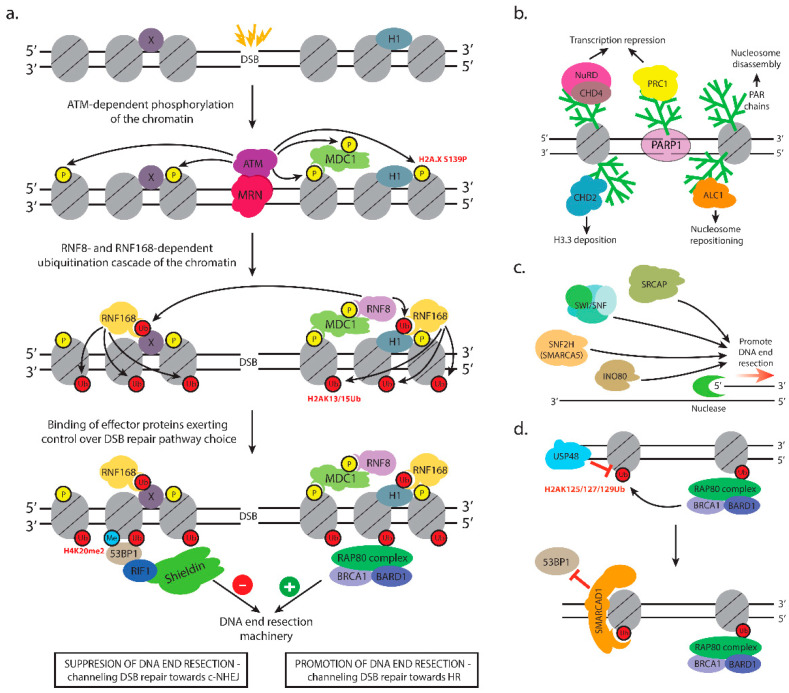
Chromatin response to double-strand DNA breaks. (**a**) Major chromatin modifications exerting control over DSB repair pathway choice. (**b**) PAR-dependent chromatin remodeling following PARP1 binding to DSBs. (**c**) SRCAP, SWI/SNF, SNF2H, and INO80 lead to chromatin changes facilitating DNA end resection and HR. (**d**) BRCA1-BARD1 ubiquitinates H2AK125/127/129Ub in the vicinity of DSBs leading to the recruitment of SMARCAD1, which restricts 53BP1 binding to the chromatin surrounding the break. The deubiquitinase USP48 counteracts H2A ubiquitination by BRCA1-BARD1 promoting 53BP1 binding.

The accurate pathway choice for DSB repair is fundamental for the efficient repair of the damage. Repair pathway choice depends on many factors, such as the cell cycle phase, the nature of the damage, and the chromatin compartment where the break has occurred. Whether cells choose to execute c-NHEJ or HR on a particular DSB following damage recognition is determined by a remarkably complex array of chromatin modifications which ordain the recruitment of downstream effector proteins that are executing the repair process. The most important protein factors that exert control over DSB repair pathway choice are p53-binding protein 1 (53BP1) and BRCA1, which exhibit opposite functions with respect to initiation of 5′-end DNA resection [18]. 53BP1 opposes 5′-end resection directing DSB repair towards c-NHEJ. 53BP1 achieves this by binding to the chromatin in the vicinity of the breaks and recruiting downstream factors such as Rap1-Interacting Factor 1 (RIF1) and the Shieldin complex, which protect broken DNA ends from nucleolytic degradation [89]. On the other hand, BRCA1 interacts and activates proteins that initiate and execute extensive 5′-end resection, which is a prerequisite for DSB repair by homologous recombination [90]. Both proteins are recruited to DSBs by means of specific chromatin modifications and chromatin remodeling events executed by a multitude of chromatin-modifying DNA repair factors [91]. Next, we discuss in detail the chromatin events that define and orchestrate DSB repair (Figure 3, Table 1 and Table 2).

### 3.1. ATM and the Control of Early Events in DSB Repair

ATM is the apical kinase responsible for global orchestration of cellular responses to DSBs, which include DNA repair, checkpoint activation, alterations in chromatin structure, transcription, apoptosis, and senescence [36]. ATM is recruited to chromatin in response to DSBs [92,93] in a process that requires ATM binding to the C terminus of NBS1 [53]. MRN both recruits ATM to DNA lesions and stimulates ATM kinase activity once there [53,94,95] (Figure 3a). In a series of papers, Price et al. have unveiled the chromatin control of ATM activity in response to DSBs. To become activated, the ATM kinase needs to be acetylated by the TAT-interactive protein 60 kDa (TIP60) histone acetyltransferase (HAT). The interaction between ATM and TIP60 is not regulated in response to DNA damage, but the HAT activity is specifically activated by DNA damage [96]. TIP60 is activated by the direct interaction between the chromodomain of TIP60 and histone H3 trimethylated on lysine 9 (H3K9me3) as well as the MRN complex [97]. In turn, H3K9me3 at DSBs is regulated by a complex containing KAP-1, HP1, and the H3K9 methyltransferase Suv39h1. Price et al. demonstrate that a complex containing KRAB-associated protein 1 (KAP-1), heterochromatin protein 1 (HP1), and the H3K9 methyltransferase Suppressor of Variegation 39 Homolog 1 (Suv39h1, KMT1A) is rapidly loaded onto the chromatin at DSBs. Suv39h1 methylates H3K9 facilitating the loading of additional KAP-1/HP1/Suv39h1 through binding of HP1′s chromodomain to the nascent H3K9me3. This process initiates cycles of KAP-1/HP1/Suv39h1 loading and H3K9 methylation that facilitate the spreading of H3K9me3 and KAP-1/HP1/Suv39h1 complexes and transient formation of heterochromatin for tens of kilobases away from the DSB [97]. However, the DSB-specific increase in H3K9me3 has to be reconciled with data showing that H3K9 demethylase KDM4D was recruited to DSBs in a PARylation-dependent manner and controlled recruitment of ATM there. KDM4D deficit affected both HR repair and NHEJ [98,99]. These discrepancies may be explained by temporal differences in the recruitment of activities that increase or decrease H3K9me3.

ATM activation has been shown to depend on histone acetylation. Thus, ATM function in response to ionizing radiation was shown to depend on histone H4K16 acetylation carried out by the histone acetyltransferase Males absent On the First (MOF, KTM8) [100]. The nucleosome-binding protein High-Mobility Group Nucleosome binding 1 (HMGN1) was found to modulate the global organization of ATM throughout the nucleus via promoting DNA damage-induced H3 acetylation. Thus, HMGN1 predetermines the interaction of ATM with chromatin before induction of damage [101].

ATM phosphorylates hundreds of substrates in response to DNA damage [102], although it is unclear what proportion of these are functionally important [36] (Figure 3a). Many ATM substrates are also phosphorylated by ATR in response to replication stress. Multiple crosstalks exist between ATM and ATR as ATM-dependent DNA end resection provides the RPA-ssDNA signal for ATR recruitment and activation [36]. Recent data indicate that the outcomes of the crosstalks depend on the cell cycle phase and the damage load [103,104].

ATM controls accessibility to damaged chromatin. RNF20 and RNF40 (orthologs of the budding yeast protein BREfeldin A sensitivity-Bre1) catalyze the ubiquitination of H2B on lysine 120 [105] (monoubiquitination is mapped on H2B lysine 119 in *S. pombe*, on lysine 120 in humans and lysine 123 in *S. cerevisiae* [106]). Throughout the text these numbers are maintained. Upon DSB induction, RNF20-RNF40 is recruited at DSB sites, undergoes ATM-mediated phosphorylation, and ubiquitinates H2B. This is essential for the timely accumulation of NHEJ and HR proteins at DSB sites and subsequent proficient repair via both pathways. The depletion of RNF20-RNF40 led to reduced accumulation of YFP-tagged XRCC4 and Ku80, as well as delayed recruitment to DSBs of RPA, BRCA2, and RAD51. H2BK120ub is thought to initiate chromatin disassembly providing access to both NHEJ and HR DNA repair factors [107]. H2BK120ub did not affect γH2A.X phosphorylation [108]. Another study, however, found that the SAGA complex deubiquitinase activity was required for optimal irradiation-induced γH2A.X formation, and failure to remove H2BK120ub inhibited ATM- and DNA-PKcs-induced γH2A.X formation. These data suggest that deubiquitination is either required at specific regions or that the mark is dynamic at DSBs [109]. Accessibility at DSBs provided by H2BK120ub was dependent on the recruitment of the Imitation Switch Nuclear ATPase 2H (SNF2H)-containing chromatin remodeling activities (Figure 3c). Initially, it was thought to affect RAD51 and BRCA1 recruitment [110], but later it was found to mediate chromatin relaxation and to be necessary for both HR repair and NHEJ [111]. SNF2H recruitment and chromatin relaxation are also dependent on the deacetylation of H3K56ac by Sirtuin 6 (SIRT6), and its deficit impaired both HR and NHEJ [112].

ATM plays a key role in increasing accessibility of heterochromatin. One of ATM substrates, KAP-1, is phosphorylated at the sites of damage, which results in its spread throughout chromatin, thereby promoting relaxation on a global scale and facilitating rapid genome surveillance [113]. Goodarzi et al. [114] have provided evidence that ATM has a specialized role in the repair of DSBs in tightly compacted heterochromatic parts as it loosens the KAP-1 binding affinity for chromatin. Phosphorylated KAP-1 dispersed Chromodomain Helicase DNA binding protein 3 (CHD3) subunit of the Nucleosome Remodeling Deacetylase (NuRD) complex with concomitant chromatin relaxation [115]. ATM silences transcription by two mechanisms—the first one is dependent on RNF8 and RNF168 [116] and the other involves phosphorylation of Polybromo-associated BAF (PBAF) subunit BRG1/BRM-associated factor 180 (BAF180) and mono-ubiquitination of H2A on Lys-119 via polycomb repressive complex 1 and 2 [117]. The downregulation of transcription depends on the distance from the DSB [118].

The primary chromatin target of ATM, ATR, and DNA-PKcs is the histone variant H2A.X, and its phosphorylation is one of the earliest events following break induction [119]. A major role of γH2A.X is to spatially organize repair by the chromatin retention of repair proteins. In both yeast and mammalian cells, phosphorylated H2A.X encompasses rather extensive regions around the break—several tens of kilobases in yeast [120] and megabase-sized domains in mammalian cells [119]. The spread of γH2A.X requires MDC1—a large protein platform that supports the chromatin recruitment of many DSB repair factors (Figure 3a). MDC1 stabilizes the interaction of NBS1 component of MRN with chromatin at DSBs [55], and the MRN complex recruits more ATM [121,122]. This creates a positive feedback loop that propagates H2A.X phosphorylation [123,124]. One way by which ATM can increase MRN binding is by locally increased H3K36me2 after ATM phosphorylation abrogates chromatin binding of KDM2A demethylase [125]. γH2A.X-marked chromatin is transcriptionally inactive [126].

It has been shown that MDC1 binding to γH2A.X is inhibited by phosphorylation of tyrosine 142, which promotes pro-apoptotic factors, thus controlling life-death cell fate decisions [127]. At replication fork stalling lesions, MDC1 and Topoisomerase II Binding Protein 1 (TopBP1) help anchor ATR kinase at chromatin containing damage [128].

MDC1 plays a determining role in the interaction of phosphorylated H2A.X with its downstream partners as it acts as an interaction platform for other DDR components.

### 3.2. Setting up Chromatin for DSB Repair Pathway Choice

#### 3.2.1. RNF8-RNF168-Mediated Recruitment of Repair Factors

Different sets of events set up the anti- or pro-resection chromatin environment at double-strand breaks. As already noted, one of the MDC1 interactors is the RNF8 E3 ubiquitin ligase [129]. Together with the UBC13 E2 enzyme, RNF8 catalyzes the formation of K63-linked polyubiquitin chains [129,130,131] initially thought to target H2A histones, but now shown to be mainly of H1 linker histones [58]. H1 ubiquitination serves as a recruitment signal for RNF168, which in turn, monoubiquitinates H2A-type histones at K13/K15 in a DSB-dependent manner [59,60,132]. The priming monoubiquitination on H2A by RNF168 gets extended by RNF8 to form K63-linked polyubiquitin chains needed to interact with downstream effectors [60] (Figure 3a). RNF8 also promotes the recruitment of RNF168 via extensive chromatin decondensation by bringing the CHD4 catalytic subunit of the NuRD complex. The chromatin remodeling activity of CHD4 promotes efficient ubiquitin conjugation and assembly of RNF168 at DNA double-strand breaks. Interestingly, RNF8-mediated recruitment of CHD4 and subsequent chromatin remodeling were found to be independent of the ubiquitin-ligase activity of RNF8, suggesting that CHD4 and RNF8 cooperate in creating a chromatin environment that is permissive to repair [133].

53BP1 recognizes H2AK15ub with its ubiquitin-dependent recruitment (UDR) motif [132]. Chromatin retention of 53BP1 requires histone methylation in addition to ubiquitylation. The two methylated lysines associated with retention of 53BP1 to DSB-flanking chromatin are H3K79me and H4K20me, with the latter thought to have a major role in the process [134,135,136]. Both H3K79 and H4K20 methylation are not induced in response to DNA damage. This has led to the suggestion that chromatin changes in response to damage get the chromatin marks exposed to mediate 53BP1 binding. While it is generally accepted that H4K20 methylation is not damage induced, there are data indicating that it may be increased locally upon induction of DSBs by the multiple myeloma SET (MMSET) methyltransferase. Downregulation of MMSET significantly decreased H4K20 dimethylation and subsequent accumulation of 53BP1 at DSBs [137]. Still, mice knocked-out for MMSET show normal recruitment of 53BP1 [138]. H4K20 methylation is catalyzed by several enzymes—monomethylation is carried out by SET Domain Containing 8 (SETD8, PR-SET7, KMT5a) [139,140], while the homologs SUV420H1 and SUV420H2 catalyze the di- and tri-methylation of lysine 20 on histone H4 (H4K20) and require H4K20me1 as substrate [141,142]. No major requirement for the known H4K20 (di-)methylases SUV420H1 and SUV420H2 in 53BP1 recruitment or DSB repair function was found, but the H4K20 monomethylase, PR-SET7 (SET8), was essential [138].

Histone H4K20 methylation is subject to binding by several factors, which can compete with 53BP1 for chromatin recruitment. In response to DNA damage, one of these—Lethal(3)Malignant Brain Tumor-Like Protein 1 (L3MBTL1), which binds H4K20me2 [143]—gets evicted from chromatin [144]. Another competitor is the histone demethylase KDM4A (which binds to H4K20me3) [145], which is targeted for degradation in RNF8- and RNF168-dependent manner after damage induction [146]. The H4K20me2 mark is required for the binding of the TIP60 complex as well, and TIP60 directly competes for binding with 53BP1. TIP60 is able to acetylate H2AK15 on H2A, which is mutually exclusive with K15 ubiquitination (needed for 53BP1). This establishes another switch mechanism for pathway choice [147]. In addition, Tudor interacting repair regulator (TIRR) was shown to bind to the tandem Tudor domains of 53BP1, and both stabilize 53BP1 and impede its binding to H4K20me2 when no damage is present [148]. Following ATM-mediated phosphorylation of 53BP1 after damage induction, TIRR dissociates from 53BP1, allowing its binding to H4K20me2 in the vicinity of the break.

The other histone mark implicated in 53BP1 recruitment, H3K79me [134,149], is deposited by mammalian DOT1 Like protein (DOT1L) [150,151]. Efficient H3K79 methylation was shown to depend on H2BK120 ubiquitination as it directly stimulated DOT1L activity [152,153,154]. In yeast, Dot1 and Rad9 (53BP1 homolog) also contribute to HR via loading of cohesin at sites of damage [155] as well as the repair of other types of damage [156].

The RNF8-RNF168 ubiquitination was reported to recruit BRCA1-BARD1 [61,62,63] (BRCA1-C), the key mediator of 5′-end resection. BRCA1-BARD1 interacts with CtIP and MRN in a cell cycle-dependent manner to stimulate MRE11′s activity and initiate resection [64]. However, recruitment of the Brca1-CtIP-MRN complex was found dependent on ATM and PARylation but not on RNF8/RNF168 mediated ubiquitination [157]. The BRCA1/BARD1 complex was also found to be recruited in the vicinity of the DSBs through BARD1 interaction with K9-dimethylated histone H3 (H3K9me2) mediated by heterochromatin protein 1 (HP1) [157].

At the same time, RNF8/RNF168-mediated K63-linked ubiquitin chains recruit the BRCA1-A complex [62], which consists of BRCA1/BARD1, RAP80, Abraxas, MERIT40, BRCC36, and BRCC45 [62]. Unlike BRCA1/BARD1 (BRCA-C) complex, this one limits DNA end resection [158,159] via BRCC36-mediated cleavage of K63-linked chains [160]. Kinetic data by Aleksandrov et al. obtained by micro-irradiation in living cells clearly show that BARD1 accumulation follows a similar pattern of accumulation as ubiquitin and is recruited much later compared to PARP1 [161]. As of now, the contribution of BRCA1-related pro- vs. anti-resection activities recruited by RNF8-RNF168 H2A ubiquitination remains an open question.

#### 3.2.2. BRCA1-BARD1-Dependent Repositioning of 53BP1 Promotes DNA End Resection

Other factors influence BRCA1 recruitment to DSBs as well. The BRCA1/BARD1 recruitment depends on the recognition of histone H4 unmethylated at lysine 20 (H4K20me0) by the ankyrin repeats domain of BARD1. BARD1 mutations disabling H4K20me0 recognition abrogated accumulation of BRCA1 at DSBs and allowed the anti-resection activity to prevail in S and G2 phases [162]. Methylated histones dilution following DNA replication facilitates BRCA1 recruitment to sister chromatids. Conversely, the recruitment of 53BP1 is dependent on H4K20me2, which guides it to pre-replicative chromatin. [163,164]. The histone variant macroH2A1, together with the H3K9 methyltransferase PRDM2, was required for BRCA1 recruitment [165]. This report, however, needs to be reconciled with an earlier one linking macro H2A1 with 53BP1 recruitment [166].

Super-resolution microscopy performed to examine the spatial distribution of BRCA1 and 53BP1 proteins within a single irradiation-induced focus indicated progressive BRCA1-dependent exclusion of 53BP1 from DNA damage sites during S-phase [167]. Follow-up studies provided insights on a chromatin-based mechanism dependent on BRCA1-BARD1 E3 ubiquitin ligase activity that mediates 53BP1 eviction. BRCA1-BARD1 ubiquitinates [168,169,170] lysines 125, 127, and 129 on H2A [171]. Inactivation of BRCA1-BARD1 E3 ubiquitin ligase activity impaired end resection. The SMARCAD1 remodeler has been shown to bind BRCA1-BARD1-ubiquitinated H2A [172]. SMARCAD1 and its homolog in yeast Fun30 are large, single-subunit nucleosome remodelers, which act in homodimeric form [173]. The role of Fun30 and SMARCAD1 during repair of DNA DSBs by homologous recombination has been reported by several groups [174,175,176] demonstrating a molecular function in counteracting the resection inhibitor 53BP1 [172,174,177] (Figure 3d). In the absence of 53BP1/Rad9, the remodeling activity of SMARCAD1/Fun30 is at least partly dispensable, and phenotypes such as camptothecin sensitivity are suppressed [174,175]. Both the SMARCAD1 ATPase activity and the integrity of its ubiquitin-binding domains are required for the eviction of 53BP1 to stimulate HR repair [172]. These data point to ubiquitin-targeted SMARCAD1 remodeling, rather than competition between 53BP1 and BRCA1 in 53BP1 repositioning and repair choice [172].

SMARCAD1, as well as yeast Fun30, are phosphorylated by cyclin-dependent kinase 1 [177,178] to promote interaction with the N-terminal BRCT repeats of TopBP1 and yeast Dpb11 [177]. The negative regulation to SMARCAD1 involves ubiquitin-specific peptidase 48 (USP48)—a deubiquitinase specific for BRCA1-BARD1 ubiquitination sites (H2AK125/K127/K129) [179] (Figure 3d). Overexpression of USP48 restricted resection, whereas the USP48 deficit resulted in the repositioning of 53BP1 further away from the damage site and extended resection. Cells depleted of USP48 develop a dependence on SSA even though they display normal levels of 53BP1 [179]. The depletion of compacting methyltransferases SETDB1 and Suv39, as well as HP1, prevents changes in irradiation-induced foci and inhibits HR repair [180].

### 3.3. Recruitment of Effectors

#### 3.3.1. Chromatin Changes that Promote c-NHEJ

Multiple chromatin changes contribute to efficient c-NHEJ, mostly by modulating chromatin accessibility at the sites of damage. DSB induction in human cells caused local nucleosome disassembly in G1. It encompassed a region of 2 kb around the break and was independent of DNA end resection. ATM and the INO80 chromatin remodeler promoted nucleosome disassembly (manifested by removal of histone H3 from the chromatin) during non-homologous end joining [228]. c-NHEJ also appears to be stimulated by the deposition of histone variants that destabilize the nucleosome [242]. It has been reported that the p400 remodeler deposits H2A.Z required for the loading of Ku70/Ku80 [232]. This result is in agreement with an earlier report that yeast SWR1 remodeler participates in yeast c-NHEJ by facilitating the recruitment of the Ku proteins [231]. Another histone variant that stimulates c-NHEJ is histone H3.3. Luijsterburg et al. described a mechanism initiated by PARP1, which recruits the chromatin remodeler CHD2 (Figure 3b). The remodeler, in turn, triggers rapid chromatin expansion and the deposition of histone variant H3.3 at sites of DNA damage. H3.3 deposition promotes efficient assembly of c-NHEJ complexes [235].

SWI/SNF-mediated chromatin remodeling also contributes to mammalian c-NHEJ. The BAF and PBAF complexes include one of two mutually exclusive DNA-dependent ATPases—BRG1/SMARCA4 or BRM/SMARCA2. Together with core and accessory subunits, these ATPases function in mobilizing nucleosomes to regulate transcription, DNA replication, and higher-order chromatin dynamics [243,244]. The BAF complex is needed for efficient c-NHEJ [210], as it controls the accumulation of GFP-tagged Ku70. The amount of Ku70 at laser-irradiated sites was severely reduced in cells after depletion of BRM, BAF47, BAF60a, BAF60c, BAF155, BAF250a, or BAF250b [210]. In turn, SWI/SNF remodeling to recruit Ku70 and Ku80 was dependent on p300 and CREB-binding protein (CBP) acetyltransferases (targeting H3K18, and H4K5, 8, 12, and 16), which were recruited at DSBs. Both RNA interference and HAT inhibitors anacardic acid (inhibits PCAF, p300, and TIP60) and curcumin (which inhibits CBP and p300) produced similar defects in the recruitment of Ku proteins [189].

The SAGA complex was found necessary for c-NHEJ as well. It mediated a switch of H2BK120 from ubiquitinated to acetylated state. The change in the post-translational modifications at this residue promoted histone H1 eviction and 53BP1 accumulation over γH2A.X containing domains [109,187].

Histone acetylation during c-NHEJ appears to be highly dynamic. After break induction, mammalian HDAC1 and HDAC2 are quickly recruited to DSBs and deacetylate H3K56 and H4K16. HDAC1 and HDAC2 knockdown impaired c-NHEJ [245]. The authors suggested that chromatin compaction is needed to keep Ku proteins concentrated at DSB ends and prevent them from sliding away (as they do on naked DNA) [245]. Other HDACs are required for efficient c-NHEJ: knockout of SIRT7 in mice led to an increase in H3K18ac, which impaired c-NHEJ activity [246]. SIRT6 HDAC forms a complex with DNA-PKcs. In response to DSBs, SIRT6 causes an acute decrease in global H3K9ac levels, which stabilize DNA-PKcs at chromatin adjacent to a site-specific DSB [247]. Histone deacetylases control each other. An interplay between SIRT1 and HDAC1 was shown in neurons in which SIRT1 deacetylates and activates HDAC1. HDAC1 targets H4K16ac and promotes c-NHEJ [193]. Other histone marks are reported to modulate c-NHEJ. Metnase-induced H3K36me2 enhanced the recruitment of Ku70 and NHEJ in mammalian cells [204]. Similarly, in yeast Set2-dependent H3K36 (mono-, di-, and tri-) methylation reduced resection and promoted c-NHEJ, while antagonistic Gcn5-dependent H3K36 acetylation increased resection and promoted HR. [248]. These data, however, are in contrast to findings in mammalian cells in which H3K36me3 stimulated HR via Lens Epithelium-Derived Growth Factor (LEDGF), which was found to bind CtIP [206] or TIP60 to promote H4K16ac [249]. However, while these studies suggest H3K36me3 as a DSB repair specific mark, a recent genome-wide study of repair-associated histone modifications found only a modest increase in H3K36me3 at c-NHEJ-repaired break sites [187]. It has been suggested that H3K36me3 might contribute to HDAC1/2 recruitment in order to compact chromatin structure away from the break to serve as a barrier to resection [250].

Phosphorylation of histone H4 at S1 by the casein kinase II has been shown to participate in c-NHEJ in yeast [184]. The mark inhibited NuA4 ability to acetylate H4 [183]. In mammalian cells, phosphorylated H4S1 accumulates at DSBs [187].

PARP1 has been shown to limit the resection process. Inhibition of PARP1 led to hyper-resected DNA DSBs. This phenotype was associated with loss of Ku, 53BP1, and RIF1 from the break site. EXO1-mediated resection was blocked by PARP1 [251]. Contrary to this finding, a recent report showed that the PARP1/2 inhibitor BMN673 (talazoparib) only delays the onset of 5′-end resection in living non-Hoechst-pre-treated cells possibly due to the delay in PCNA-dependent DNA repair pathways in the vicinity of the DNA break. In addition, Ku, 53BP1, BARD1, RPA, and RAD51 were recruited to laser-induced DNA damage sites in the presence of BMN673, albeit some of them with altered kinetics [161]. The recruitment of PARP1 has been shown to be limited by the histone acetyltransferase p400. The deficit of p400 increases the frequency of alternative end joining events, generating large deletions following repair of double-strand breaks [252].

#### 3.3.2. Chromatin Events Promoting the Recruitment of HR Repair Factors

The presence of nucleosomes has been shown to impede resection by both the Exo1- and Sgs1-Dna2-dependent mechanisms [253]. The authors of the study showed that the Sgs1–Dna2 machinery does not require extensive remodeling to initiate resection, which is consistent with previous in vivo data that Remodeling Structure of Chromatin (RSC) remodeling is necessary for the removal of a single nucleosome next to a site-specific DSB [254]. Exo1, on the other hand, was inhibited even when a single nucleosome was surrounded by large tracts of free DNA [217].

ChIP studies in yeast generally indicate nucleosome loss in the immediate vicinity (~500 bp) of a DSB. Histone density is much less affected within 1 kb of the lesion [120,224,231,255]. In a similar manner, data in human cells do not support significant nucleosome loss [187], although resection proceeds to about 3.5 kb from a sequence-specific defined DSB [256]. At the same time, nucleosomes bind resected DNA, and this may even stimulate pro-resection activities. Complexes between ssDNA and recombinant histones have been reconstituted biochemically. Fun30—a key remodeler linked to reversing the 53BP1/Rad9 block to resection, binds preferentially to these complexes over conventional nucleosomes, which activated its ATPase activity [217].

The effect of histone variant H2A.Z on resection is a common theme of several studies. Biochemical and genetic evidence reveal that nucleosomes harboring H2A.Z are more accessible to Exo1 [253]. This might explain the role of the transient incorporation of H2A.Z at DSBs [226,232]. In mammalian cells, the deposition of H2A.Z at damage sites was also linked to increased resection. When H2A.Z removal by Acidic Nuclear Phosphoprotein 32 Family Member E (Anp32e)—an H2A.Z chaperone—was blocked, nucleosomes at DSBs retained elevated levels of H2A.Z. This resulted in increased CtIP-dependent end resection, accumulation of single-stranded DNA, and an increase in repair by aEJ [257]. Other authors found that the deficit of mammalian SRCAP resulted in impaired resection due to defective CtIP recruitment [230] (Figure 3c). Snf2-Related CREBBP Activator Protein (SRCAP) is the mammalian homolog of yeast Swi2/Snf2-related 1 (Swr1), a remodeler that facilitates the exchange of H2A with H2A.Z variant. However, while yeast Swr1 has been shown to stimulate resection [253], an earlier study found defective Ku80 loading but normal end resection in an Swr1 mutant [231]. In addition, in both yeast and mammals, Ino80-deficient cells were found defective in end resection [222,223] (Figure 3c). If the phenotypes were determined by H2A.Z, the opposite would be expected as Ino80 and Swr1 have opposing roles in H2A.Z deposition [258,259].

Fun30 also influences the distribution of H2A.Z genome-wide and particularly in centromeric, pericentromeric, and subtelomeric chromatin [218,219]. However, it is presently unclear if these changes might potentially contribute to resection regulation.

LEDGF protein is constitutively associated with chromatin through its Pro-Trp-Trp-Pro (PWWP) domain, which binds to H3K36me3. LEDGF interacts with CtIP in a DNA damage-dependent manner, thereby enhancing CtIP tethering to the active chromatin and facilitates its access [208]. Several studies have shown that H3K36me3 (associated with active transcription) and the SETD2 methyltransferase are required for HR repair [205,206,207].

In yeast, the absence of functional SWI/SNF activity impaired the recruitment of MRX and significantly delayed the initiation of DNA end resection [216]. The human Helicase, Lymphoid Specific (HELLS) protein—an Snf2-like chromatin remodeler, dysregulated in several cancers—was shown to promote the accumulation of CtIP at IR-induced foci. It contributed to repair within heterochromatic regions during the G2 phase, suggesting that it might be required for efficient repair in specific genomic contexts [260]. CHD1 chromatin remodeler has been shown to be required for the recruitment of CtIP. CHD1 depletion sensitized cells to PARP inhibitors [233].

RAD51 nucleoprotein filament formation is stimulated by several chromatin factors. Asf1 and CAF-1 histone chaperones are needed for RAD51 loading during HR repair. The knockdown of Asf1 or CAF-1 reduced the recruitment of the RAD51 loader MMS22L-TONSL to ssDNA. Deficient cells had persistent RPA foci, extensive DNA end resection, and persistent activation of the ATR-Chk1 pathway [261]. The INO80 chromatin remodeler has also been implicated in stimulating RAD51 binding to resected DNA during HR repair. The authors delineated two distinct functions of the INO80 remodeler during HR—DSB end resection (see above) and presynaptic filament formation. The latter function depended on INO80-mediated removal of H2A.Z from damage sites, which promoted RAD51 loading [227]. A similar function has been reported for the p400 remodeling complex. p400 was found important for the recruitment of RAD51 to DSB sites and for survival after DNA damage. p400 and RAD51 were present in the same complex, and both favored chromatin remodeling around DSBs [262].

Data in yeast indicated that the SWI/SNF chromatin remodeler is recruited to HO endonuclease-induced DNA break site and participates in HR repair by remodeling nucleosomes at the donor locus, while another remodeler, RSC, is required following synapsis to complete the repair event [214].

Depletion of BRG1 (the catalytic subunit of mammalian SWI/SNF complexes) impaired HR repair as measured using reporter constructs specific to this repair pathway. The defect was attributed to defective RAD51 filament assembly and increased RPA retention, suggesting that BAF and/or PBAF may specifically promote the exchange of RPA with RAD51 on ssDNA [263].

Chromatin factors also modulate HR repair machinery via transcriptional effects. The histone lysine demethylase PHD finger protein 2 (PHF2) is deregulated in various cancers. PHF2 knockdown decreased CtIP and BRCA1 protein and mRNA levels. The authors also suggested that it might interfere with H4K20me2 and 53BP1 recruitment to DNA lesions [264]. In addition to its direct roles at DSBs, the NuRD complex affects RAD51 abundance transcriptionally. CHD4, a component of the complex, has recently been shown to be required for proper RAD51 gene transcription [265].

#### 3.3.3. Chromatin Compaction in HR Repair

A number of chromatin changes that promote compaction are necessary for efficient HR repair.

The NuRD remodeling complex has been implicated in promoting HR repair by repressing transcription at DSBs [237,238] (Figure 3b). The complex has also been linked to the recruitment of a number of repair proteins, including BRCA1 [239]. The depletion of the NuRD subunit CHD4 affects the phosphorylation of the ssDNA-binding complex RPA, which is indicative of a defect in DNA end resection [266]. The association of NuRD itself to DSBs was found to depend on PARP activity [238,239,267]. More recent studies have established that NuRD is recruited to DNA breaks by Zinc Finger MYND Domain-Containing Protein 8 (ZMYND8) [268]. ZMYND8 contains a BRD domain, which binds to N-terminally acetylated histone H4 (mediated by TIP60), as well as a PHD and PWWP domains [269]. The N-terminal PHD-BRD-PWWP domains of ZMYND8 engage chromatin through multivalent interactions [270]. It has been reported that H3K4me3 demethylation near DSB sites carried out by the KDM5A demethylase is required for ZMYND8-NuRD binding to the lesions [198]. ZMYND8 binding is spatially controlled by another bromodomain protein—BRD2. BRD2 was found to be spatially restricted to a chromatin domain extending only 2 kb on either side of the DSB, and ZMYND8 spreads along the flanking chromatin but is excluded from the BRD2 region [271].

Another factor repressing transcription to promote HR repair is H2AK119 ubiquitination [195]. B Lymphoma Mo-MLV Insertion Region 1 protein (BMI1) and RNF2, as components of the Polycomb repressive complex 1 (PRC1), accumulate at DSB sites and carry out DNA damage-dependent H2AK119 monoubiquitination [272]. The major role of H2AK119 monoubiquitination is to transcriptionally silence genes. A reporter system visualizing transcription at DSB sites has shown that it was silenced locally following DSB induction [116]. Inhibition of both RNA polymerase I and II at DSB sites depended on RNF2/BMI-dependent monoubiquitination of H2AK119 [272] (as well as on ATM [273]). H2AK119ub was also dependent on PBAF (but not BAF complex) [117]. Surprisingly, the reversal of H2AK119ub by the BAP1 deubiquitinase was reported to promote homologous recombination. The authors suggested that the mark might be dynamic at damage sites [196]. The NuRD complex might also be involved in reversing H2AK119 ubiquitination. NuRD has been reported to interact with the deubiquitinase USP11 and to perform coordinated deubiquitination (by USP11) and deacetylation (by NuRD) at a DSB site [274].

Surprisingly, PRC1-mediated transcriptional repression also appears to stimulate c-NHEJ. Both BMI1 and its interactor eleven-nineteen leukemia protein (ENL)—a transcription elongation factor that is phosphorylated by ATM following DNA damage [202]—were required for the accumulation of Ku70 at DSBs; however, it is unclear if this is linked to PRC1-mediated transcription repression there. It has been suggested that silencing might be needed for retention of c-NHEJ factors, as transcription machinery could evict them [275]

It has been found that the PRC2 complex is also required for DSB-induced silencing of transcription and stimulation of repair [117]. Enhancer of Zeste Homolog 2 (EZH2) methyltransferase, a component of PRC2, is brought to sites of damage via the chromodomain Y-like protein 1 (CDYL1). CDYL1 recruitment to DSBs was reported to be PARP1-dependent, and its deficiency impaired HR repair [203]. Localization of EZH2 at DSBs by this mechanism increased the H3K27me3 repressive mark at DSBs.

H2A.Z, which, as discussed above, might promote resection, also represses transcription when it becomes incorporated into a gene body (but not when it is incorporated at a promoter [276]), suggesting that it leads to transcription silencing if a DSB arises in gene bodies.

It should be noted, however, that while chromatin factors silence transcription at DSBs, multiple recent reports suggest accumulation of RNA polymerase II at DSBs and initiation of transcription there. These DNA damage response RNAs regulate the early recruitment of crucial DSB signaling factors, including MDC1, RNF168, 53BP1, and RIF1, as well as the formation of K63-linked ubiquitin moieties, and future studies will have to reconcile the various aspects of transcriptional regulation in DSB repair [275].

### 3.4. Dynamics of DSB Repair in Living Cells

Although highly informative regarding the functions of individual DNA repair proteins and the interactions between them, in vitro studies cannot provide information about the exact time scale of DSB repair factor binding to damage sites. The temporal and spatial coordination between different DNA repair pathways can only be obtained by live-cell methods, such as live-cell imaging of the recruitment of fluorescently-labeled DNA repair proteins at laser-induced DNA damage sites [277,278,279]. A recent study reported the kinetics of recruitment and removal of 70 DNA repair proteins at laser-induced DNA damage sites in living HeLa Kyoto cells, including numerous proteins involved in DSB repair [161]. PARP1 and PARP2 are among the first proteins to be recruited to DNA damage sites (with halftimes of recruitment in the range between 2.5–5 s), and their binding to DNA lesions is required for the efficient and timely recruitment of downstream repair factors. HDAC1 and HDAC2 were also amid the fastest proteins recruited, with halftimes between 3.5 s and 5 s, indicating the need for rapid histone deacetylation following DNA damage induction. The chromatin remodeler proteins SPT16 (a subunit of the FACT complex), SNF2H (SMARCA5), and SMARCAD1 are recruited shortly after PARP1/2 and HDAC1/2 with halftimes of recruitment between 8 s and 23 s. These proteins, as discussed, have been implicated in the early chromatin remodeling events around DSBs. Early responders to DSBs, such as Ku70, LIG4, RAD50 (MRN complex), ATM, MDC1, and notably the histone chaperone CAF-1, were recruited immediately after the chromatin remodelers with halftimes of recruitment between 23 s and 60 s. These were followed by the binding of the RNF168 ubiquitin ligase (halftime of 78 s), which, as already discussed, is part of a complex ubiquitination cascade of the chromatin in the vicinity of the break that is responsible for the recruitment of downstream effector proteins. Interestingly, it was found that ubiquitin, which is recruited with a halftime of about 180 s, is the most abundant protein at laser-induced damage sites, highlighting once more the important role of chromatin ubiquitination in the organization and orchestration of DNA repair. Proteins implicated in the DSB repair pathway choice, such as 53BP1, PTIP (PAXIP1), RNF169, and BARD1, were recruited to DNA damage sites following the extensive ubiquitination of the chromatin with halftimes of recruitment between 200 s and 300 s. RPA1, s subunit of the RPA complex, RAD17, a specific subunit of the RAD17-RFC2-5 clamp loader complex, and RAD1, a subunit of the RAD9-RAD1-HUS1 DNA clamp that is loaded onto ssDNA by the RAD17-RFC2-5 complex and regulates resection, are recruited simultaneously (halftimes of recruitment between 600 s and 700 s) to laser-induced DNA damage sites with their presence indicating the implementation of 5′-end resection [280,281]. The latest proteins to be recruited (halftimes of over 900 s) are RAD51 and its associated factors, such as RAD51AP1 and RAD54B, which participate in the late steps of DSB repair by HR. Such type of studies can answer many pressing questions regarding the chronology and coordination of the numerous chromatin events that govern DSB repair. Importantly, such a systematic live-cell imaging approach may be employed to gain an in-depth knowledge of the mechanisms of action of DSB repair-targeting drugs (as has been shown for the PARP1/2 inhibitor BMN673) [161] and assess their potency in specific genetic context (e.g., BRCA1/2-deficiency).

## 4. Targeting the Chromatin Response to DSB DNA Damage

### 4.1. A New Hope: Synthetic Lethality and PARP1/2 Inhibitors

Overcoming the current flaws of anticancer chemotherapy necessitates the development of new therapeutic paradigms that take into account the specific genetic makeup of cancer cells leading to more personalized approaches to treating cancer patients. Such a novel therapeutic approach is the notion of synthetic lethality. It combines the consequences of specific genetic mutations in cancer cells with the pharmacological inhibition of a particular cellular protein, which results in a synergistic effect between these two events leading to cancer cell death [282,283]. Synthetic lethal interactions between pairs of proteins and/or pairs of pathways are especially prominent in DDR, which offers multiple redundant and/or interdependent pathways that, if restrained simultaneously, lead to profound cancer cell death [284].

**Figure 4:**
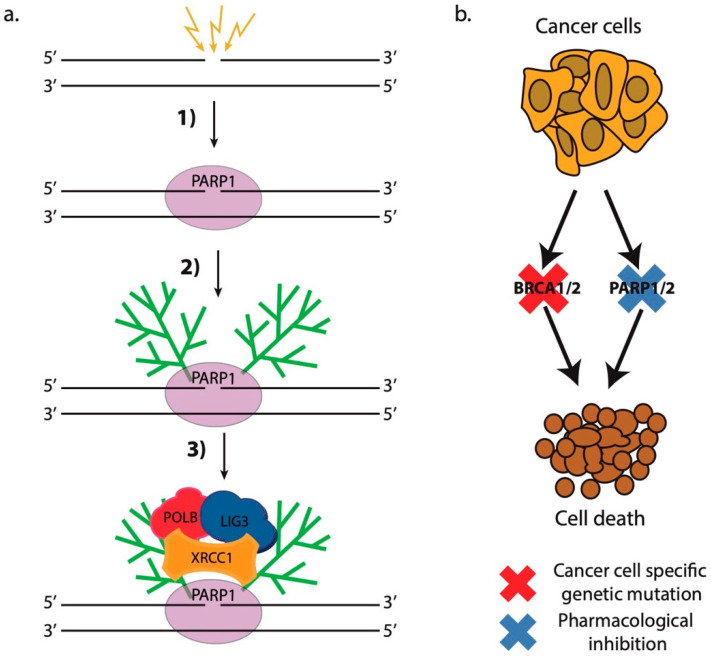
PARP1 and synthetic lethality. (**a**) (1) PARP1 binds to single- and double-strand breaks in DNA; (2) Binding to DNA lesions stimulates PARP1 activity which results in the synthesis of long negatively charged chains of poly(ADP-ribose) (PAR) on the chromatin surrounding the break and on itself; (3) Multiple PAR-binding proteins, such as XRCC1, bind to the PAR chains and repair the lesion; (**b**) Cancer cell-specific genetic defects, which are not present in normal non-cancerous cells, such as BRCA1/2 mutations, may be exploited in combination with specific pharmacological inhibitors, such as PARP1/2 inhibitors, to specifically kill cancer cells.

The first drugs that act by the principle of synthetic lethality, and currently still the only approved synthetically-lethal drugs for clinical use in cancer patients, are the PARP1/2 inhibitors (PARPis) [284]. These drugs encompass small molecules that inhibit the enzymatic activity of PARP1 and PARP2. The PARP1 protein is an essential DNA repair protein that acts as a DNA damage sensor that recognizes single- and double-strand breaks in DNA (Figure 4a) [285,286]. Following its binding to the lesion, which is very rapid in living cells [161,287], PARP1 activates its enzymatic activity, which leads to the synthesis of long, negatively charged poly(ADP-ribose) chains on the nearby chromatin proteins. These PAR chains serve as binding sites for the recruitment of downstream DNA repair factors, which actually repair the lesion [79,286,288,289]. Importantly, PARP1 and PARylation exert significant remodeling effect on the chromatin in the vicinity of DSBs that is necessary for proper DNA repair [80]. PARP1 modifies nucleosomal histones causing nucleosome disassembly, which in turn leads to chromatin relaxation [290] (Figure 3b). In addition, several chromatin remodeling complexes possess PAR-binding motifs (PBMs) and bind to DNA damage sites in a PAR-dependent manner. Such remodelers are amplified in liver cancer protein 1 (ALC1) [234], chromodomain helicase DNA-binding protein 2 (CHD2) [235], CHD4—a subunit of the nucleosome remodeling and deacetylase complex (NuRD)—and Polycomb repressive complex 1 (PRC1) [237]. Interestingly, these chromatin remodelers exhibit opposing functions with respect to the chromatin changes they induce. ALC1 repositions nucleosomes in the chromatin surrounding the break, which increases the accessibility of the damaged site to downstream factors [234], while CHD2 deposits the histone variant H3.3 in the vicinity of DSBs resulting in chromatin relaxation that promotes c-NHEJ [235] (Figure 3b).

In contrast, the NuRD and PRC1 complexes have well-defined functions as transcriptional repressors, and their recruitment to DSB sites may lead to shut down of transcription near DNA breaks, thereby preventing possible conflicts between transcription and DNA repair machinery [80,237] (Figure 3b).

PARPi have been shown to effectively kill cancer cells harboring gene mutations in BRCA1 and BRCA2 (Figure 4b) [291,292,293,294]. A significant number of breast and ovarian tumors, but also prostate and brain tumors, bear such mutations that make them prone to PARPi treatment [295]. PARP1/2 inhibitors not only inhibit PARP1 enzymatic activity, but they also change PARP1 conformation so that it becomes trapped onto the lesion following damage recognition hindering the faithful repair of the damaged region. It has been shown that PARPi cytotoxicity against BRCA1/2-deficient cancer cells correlates with their trapping potency and not with their inhibitor properties [296,297,298,299]. PARPi treatment leads to a significant rearrangement in the order of recruitment and dissociation of DNA repair proteins at complex DNA damage sites, altering the coordination of the DNA damage response and possibly the outcome of the repair process [161]. In addition, it has been shown that tumors that are defective with respect to other HR factors such as ATM, RAD51, PALB2, etc., are also prone to PARPi treatment [283,300]. Moreover, PARP1 has been implicated in the protection and stabilization of stalled replication forks due to replication stress—a function that gains extreme importance in BRCA1/2-deficient context and rationalizes once more the use of PARPi in treating BRCA-deficient cancer patients [80,301].

PARP1/2 inhibitors being superior to existing cancer treatment regimens for HR-deficient cancer patients, as clearly manifested in clinical trials, led to their approval for clinical use for the first time in 2014. Currently, there are four clinically-approved PARP1/2 inhibitors—olaparib (Lynparza^®^, Astra Zeneca), rucaparib (Rubraca^®^, Clovis), niraparib (Zejula^®^, Tesaro), and talazoparib (Talzenna^®^, Pfizer)—which, at least for now, are almost exclusively used for treating HR-deficient types of cancer or cancers that have responded well to previous therapy with platinum compounds [282,302]. As of the end of 2019, there are almost 200 ongoing clinical trials that assess the potency of the abovementioned PARPi, and many others are still not yet approved for clinical use (https://clinicaltrials.gov/). PARP1 facilitates not only the repair of single- and double-strand breaks but interacts with TDP1, which removes DNA topoisomerase I covalent complexes from DNA that are generated in the presence of topoisomerase inhibitors such as camptothecin and its derivatives (TOPccs, topoisomerase cleavage complexes) [300,303]. PARP1 associates with and PARylates TDP1, stimulating its enzymatic activity to eliminate transcription- and replication-blocking TOPccs. This justifies the most frequently used combinations used in the clinic–PARPi + temozolomide (a DNA alkylating agents) and PARPi + DNA topoisomerase I inhibitors [300].

Unfortunately, as has been shown for virtually all classes of anticancer drugs, cancer cells are able to evolve resistance. Currently, multiple mechanisms that could lead to PARPi resistance have been described, including BRCA1/2 reversal mutations [304,305], loss of non-homologous end joining factors such as 53BP1 [306], RIF1 [307,308] and subunits of the Shieldin complex [309,310,311,312], mutations in the PARP1 protein itself [313], loss of DYNLL1–an end resection inhibitor protein [314], and probably many more yet to be discovered [315]. All these resistance mechanisms heavily involve the chromatin landscape in the vicinity of the breaks, and understanding the dynamics of chromatin changes during DSB repair may provide novel avenues for combating PARPi resistance in the clinic.

PARP1 and BRCA1/2 is not the only synthetically lethal pair of genes linked to DSB repair that has been described. Another such pair is formed between Pol Θ and BRCA1/2. As described above, Pol Θ is a DNA polymerase that is essential for alternative end joining [316,317]. aEJ becomes a critical pathway for DSB repair in HR-deficient cells harboring mutations in BRCA1/2 (and possibly other HR genes); therefore, Pol Θ inhibition or knockdown significantly reduces cell survival in a BRCA1/2-deficient context [83,84]. The SWI/SNF chromatin remodelers are huge protein complexes that are vital for reorganizing chromatin in processes such as transcription, replication, and DNA repair [243]. Tumors harboring mutations in SNF5—a subunit of the SWI/SNF complex that exhibits transcriptional co-activator properties—are profoundly sensitive to the inhibition of EZH2—the enzymatic methyltransferase subunit of the PRC2 complex that represses active chromatin and drives epigenetic silencing. EZH2 leads to suppression of p16—a prominent tumor suppressor gene—in SNF5-deficient cancer cells [318]. Moreover, EZH2 levels are increased in SNF5-deficient cells. Hence, EZH2 inhibitors (EZH2i), such as EPZ-6438, have been shown to abrogate EZH2 activity and reactivate p16 expression in SNF5-deficient context both in vivo and in vitro significantly inhibiting their proliferation [318,319,320]. These results prompted the design and implementation of clinical trial studies to investigate the efficiency of EZH2i in treating SNF5-deficient cancer patients (https://clinicaltrials.gov). Undoubtedly, with the advancement of more elaborate functional genomics approaches such as CRISPRi screens, more synthetically lethal pairs of genes will be uncovered, driving the development of a new generation of anticancer drugs that are more effective and tailored to the specific mutational context of the cancer patients [284].

### 4.2. Targeting Chromatin Regulators

The complicated nature of the chromatin response to DSBs offers multiple possibilities for drug targets. In the following paragraphs, we provide examples of targeting the chromatin regulators that participate in DSB repair.

Early studies of ATM and ATR have shown that caffeine sensitizes cells to genotoxic agents [321,322] and those with p53 defects were particularly sensitive [323,324,325]. However, the doses required for radiosensitization cannot be achieved in patients [326]. Similarly, wortmannin, a pan-PI3K inhibitor [327], is a potent radiosensitizer [328], but lacks selectivity and has too high in vivo toxicity [329] to be useful as a drug.

The first potent and selective ATM inhibitor was KU-55933. KU-55933 sensitized cancer cells to DSB-inducing chemotherapeutics, such as the topoisomerase II inhibitors etoposide and doxorubicin as well as ionizing radiation. In cells without functional ATM, radiosensitization was not observed [330]. A similar inhibitor, KU-60019, sensitized to irradiation highly resistant glioblastoma cell lines [331,332]. KU59403 was the first compound to achieve tissue distribution with concentrations exceeding those required for in vitro activity. It caused significant chemosensitization in in vivo models of human cancer [333].

ATM is also increasingly being recognized as a target for synthetic lethality with PARP inhibitors. The ATM inhibition combined with PARP1 depletion or inhibition caused a massive synergistic increase of DNA damage suggesting the effectiveness of combined treatments [334].

Very few tumors harbor ATR mutations. Increasing evidence indicates that high levels of replication stress in tumors make them particularly vulnerable to the loss of ATR [335].

In spite of concerns that ATR inhibition could cause severe side effects in highly proliferative normal tissues, studies suggest that ATR inhibition might be cytotoxic specifically to cancer cells, especially in p53-defective backgrounds [336,337]. The reduction of ATR expression to 10% of normal levels was synthetically lethal in Ras-driven tumors, while normal bone marrow and intestinal cells were minimally affected [338]. Schisandrin B, a naturally-occurring compound and the active ingredient of Fructus schisandrae, was reported as a selective inhibitor of ATR [339]. A compound called VE-821 was shown to be a potent inhibitor of ATR with minimal cross-reactivity (>600-fold selectivity over ATM or DNA-PKcs) [340]. It showed strong synergy with genotoxic agents and ionizing radiation in HCT116 cancer cells. Not surprisingly, synergy was most pronounced with the cross-linking drugs cisplatin and carboplatin [341]. A related compound, VE-822, markedly increased the sensitivity of ATM-deficient lung adenocarcinoma cells leading the authors to propose that ATM deficiency could be useful as a marker for ATR and PARP sensitivity [342]. In a cellular model of oxaliplatin resistance, VE-822 successfully overcame the drug resistance [343].

AZD6738, an orally available inhibitor of both ATR and Chk1 [344], showed synthetic lethality in various cancer cell lines [345,346], including in combination with the WEE1 inhibitor AZD1775, causing mitotic catastrophe in triple-negative breast cancer cell lines [347].

An ATR inhibitor, AY1895344, developed by Bayer, showed strong monotherapy efficacy in cancer xenograft models that carry DNA damage repair deficiencies and a strong synergistic anti-proliferative activity in vitro in combination with olaparib [348].

NU5455, a novel highly selective oral inhibitor of DNA-PKcs, increased the efficacy of a parenterally administered topoisomerase inhibitor in liver tumor xenografts without inducing any adverse effects. It augmented the effect of targeted radiotherapy of lung tumors and helped to avoid inflicting acute DNA damage and toxicity to normal surrounding tissues [349]. For a detailed review of PI3K inhibitors, including natural compounds, the reader is referred to [350].

Histone deacetylase inhibitors (HDACi) are also regarded as promising anticancer drugs. They were initially described as compounds that induced tumor cell differentiation [351,352]. The effects of HDAC inhibition depend on altered histone acetylation or acetylation of DSB repair factors, but mostly on expression changes. Trichostatin A (TSA) and suberoylanilide hydroxamic acid (SAHA) belong to the same group of pan-HDAC inhibitors and have been shown to downregulate DSB repair factors [353,354,355,356] with concomitant radiosensitization. Other HDAC inhibitors—valproic acid [357], panobinostat [358], MS-275 [359], and entinostat [360]—inhibited the expression of DSB repair and signaling proteins sensitizing cellular models of various tumors.

HDAC inhibitors also affect the DNA damage response in non-transcription-related ways. TSA treatment significantly increases PARP1 binding to DSBs, resembling PARP “trapping.” The knockdown of PARP1 mitigated the inhibitory effect of HDACi on c-NHEJ. These results indicate that a combination of HDACi and PARP1 inhibition might augment the cytotoxicity in leukemia cells by impairing c-NHEJ [361]. Sirtuin-type HDACs affect HR repair by directly deacetylating resection machinery—SIRT1 deacetylates NBS1, which stimulates HR repair [362]. As already noted, TSA and sodium butyrate led to longer persistence of GFP-tagged Ku70 and Artemis at micro-irrigation sites [245].

HDAC3 inhibition using a first-in-class selective inhibitor, RGFP966, resulted in increased apoptosis in cutaneous T cell lymphoma cell lines. These effects were associated with DNA damage and impaired S-phase progression. Isolation of proteins on nascent DNA (iPOND) indicated that HDAC3 associated with chromatin around replication forks and its inhibition resulted in a significant reduction in DNA replication fork velocity [363].

HDACi are used to treat other diseases as well [364,365] and their application should go along with the understanding that they disrupt genome integrity and may promote cellular transformation [366,367,368,369].

Several known compounds of plant origin inhibit histone acetyltransferases. Anacardic acid is a bioactive phytochemical, structurally related to salicylic acid, isolated from the shell of *Anacardium occidentale* nuts. It is a noncompetitive inhibitor of p300, PCAF, and TIP60 [370,371], which are involved in both HR and c-NHEJ [189,192,372]. As expected, anacardic acid sensitizes cells to ionizing radiation [373]; however, its low cell permeability limits its practical applications [374]. Garcinol targets p300 and PCAF [375] causing radiosensitization [376], and curcumin, found in the spice turmeric, is a specific inhibitor of CBP and p300 [377]. All three inhibited c-NHEJ when assessed by a chromosomally integrated reporter construct [189,378]. Curcumin also reduced the expression of BRCA1 by changes to the BRCA1 promoter and was also shown to inhibit the ATR kinase [378]. Due to poor bio-absorption, several analogs of curcumin have been developed. One of these analogs—H-4073, enhanced the therapeutic efficacy of cisplatin [379]. A novel low molecular weight compound, C646, is a selective HAT inhibitor that binds p300 and induces apoptosis in prostate [380] and gastric [381] cancer cell lines.

Targeting enzymes that mediate methylation in the course of DSB repair is significantly less explored. In mammalian cells, DOT1L (KMT4) methylates H3K79. The interest to develop inhibitors of DOT1L is fueled by the fact that this enzyme is a key factor in MLL-rearranged leukemia, where the MLL gene is rearranged to fuse with several different genes [382], and these fusions aberrantly recruit DOT1L enhancing the expression of genes required for pathogenesis [383]. PZ004777—a selective inhibitor of DOT1L—blocked the expression of MLL-fusion target genes, and displayed anti-proliferative effects [384]. Although the effects of this drug on DNA repair have not been directly studied, existing data suggest synergistic anti-proliferative activity in combination with chemotherapy compounds, including topoisomerase inhibitors [385]. The interest in developing novel DOT1L inhibitors is likely to increase [386], given that they might be effective in diseases other than MLL [387,388,389].

H4K20 monomethylation by SETD8 could be inhibited by nahuoic acid A—a polyketide produced in culture by *Streptomyces* sp. obtained from tropical marine sediment and the first known selective inhibitor of SETD8 [390]. It has also been reported the discovery of UNC0379, the first substrate-competitive inhibitor of SETD8, selective for SETD8 over 15 other methyltransferases [391].

Recently, A-196, a potent and selective inhibitor of SUV420H1 and SUV420H2, has been identified as a substrate-competitive inhibitor of both enzymes. In cells, A-196 induced a global decrease in H4K20me2 and H4K20me3 and a concomitant increase in H4K20me1. A-196 inhibited 53BP1 foci formation upon ionizing radiation and reduced NHEJ-mediated DNA-break repair [392]. The development of two other compounds that could markedly reduce H4K20me2 in a dose-dependent manner has been reported [393].

Another histone methyltransferase actively targeted for inhibitor development is EZH2, a subunit of the PRC2 complex, responsible for the majority of genome-wide H3K27me3 trimethylation [394]. EZH2 is overexpressed in prostate, breast, kidney, and lung cancers in which EZH2 up-regulation induces cell migration, colony formation, and genomic instability [395]. Gain-of-function mutations were also described in follicular and diffuse large B-cell lymphomas. These have led to the development of several inhibitors, such as GSK126, EPZ005687, and EPZ-6438 [395]. Although there are no data if these affect DSB repair, as already discussed, PRC2 is recruited to DSBs in a PARP-dependent way, and loss of its components sensitized cells to ionizing radiation [237,396] suggesting that EZH2 inhibitors may be effective in combination with DSB-inducing chemotherapeutics and ionizing radiation.

BMI1 (a subunit of both PRC1 and PRC2 complexes) is overexpressed in pancreatic cancer, glioblastoma multiforme, pediatric diffuse intrinsic pontine glioma, colorectal cancer, epithelial ovarian cancer, and acute myeloid leukemia [397]. It has been found that BMI1 is necessary for the maintenance of chemoresistant stem cells capable of re-establishing disease. The BMI1 inhibitor PTC596 induced apoptosis in a p53-independent manner [398]. It is unknown if it would synergize with treatments that induce DSB breaks or stall replication forks. Similarly, it would be of interest to study the combined effects of genotoxic treatments and the recently described small-molecule orally-available inhibitors of the SWI/SNF proteins Brm1 and Brg1, given the various effects of the latter in DSB repair and replication fork stability [399].

While the list of inhibitors and targets is by no means complete, it does give a sense of the vast spectrum of opportunities for therapeutic modulation of the DNA damage response. There is no doubt that the future will see more promising and, above all, clinically applicable approaches harnessing the knowledge of the chromatin response to DNA damage.

## 5. Conclusions and Future Perspectives

Double-strand DNA breaks are among the most perilous events that cells and organisms have to cope with. Accordingly, DSB repair mechanisms, which counteract the grievous consequences of DSBs, are vastly complex cellular pathways that recognize and repair this type of lesion. The DNA damage response operates in the context of chromatin, and the ever-changing chromatin environment exerts significant control over it. Proficient DSB repair is dependent on hundreds of different DNA repair proteins that act in a concerted fashion, most of which are recruited and regulated by specific chromatin changes such as histone modifications and chromatin remodeling events. Although much is known about the activities and the interactions between these proteins and the chromatin, the precise dynamics and coordination of the events during DSB repair is largely an open question. Moreover, different cells and tissues in the body or cells with specific genetic background, such as BRCA1/2-deficient cancer cells, may accomplish DSB repair in different manners that are adapted to their specific needs [400,401,402]. The advent and implication of novel techniques, such as laser micro-irradiation followed by live-cell imaging [161] or CRISPR-guided DSB induction in living cells at specific loci complemented with live-cell imaging of DNA repair protein recruitment [403], may provide crucial new knowledge and shed light on unanticipated events during DSB repair. Functional genomics approaches such as CRISPR screens may uncover novel DSB repair factors and expose interdependent sets of events in the course of DSB repair [32,312].

In the past decade, targeting of specific players in the DNA damage response has turned into a promising avenue for the introduction of novel therapies for cancer patients. The first PARP1/2 inhibitors—the first class of anticancer drugs that employ the tactics of synthetic lethality—have already hit the clinic. Many more DDR-targeting drugs are in various phases of clinical trials, such as ATM, ATR, and DNA-PKcs inhibitors. Molecules that suppress certain histone-modifying enzymes such as HDAC inhibitors have proven efficient in the treatment of various types of cancer in the clinic and are pursued as therapy in other non-cancer-related conditions. Although the specific biochemical effects of these molecules are well investigated, much less is known about their systematic impact on the dynamics and coordination of DSB repair pathways. Moreover, the efficient reorganization and implementation of DDR in cancer patients following chemotherapy with DNA damage-inducing anticancer drugs is one of the most important reasons behind the evolution of resistance, which is often seen in the clinic. Given the complexity of the DDR, many resistance mechanisms that rely on it have been described. To deal with this prominent problem and offer cancer patients more beneficial and sparing chemotherapy regimens, novel drug targets must be uncovered. Recent years have seen tremendous progress in the discovery of novel DSB protein factors and their functions, largely due to the implementation of cutting-edge high-throughput methods. CRISPR screens have identified numerous synthetically lethal pairs of genes that can be targeted in various cancer-specific contexts [284]. Tens, if not hundreds, of DDR-targeting molecules are pursued for clinical use, and there can be no doubt that at least some of them will reach the clinic carrying benefit for cancer patients.

## Figures and Tables

**Table 1:** Post-translational histone modifications associated with DSB repair.

Modification	Function	Model Organism	References
Phosphorylation			
H2A.X S139 (H2A S129)	Primary beacon of damaged chromatin, necessary for the recruitment of chromatin regulators and repair factors	*H. sapiens, M. muntjak,* *X. laevis, D. melanogaster;* *S. cerevisiae*	[119,181]
H2A.X Y142	Inhibitory to MDC1 binding, a switch between DNA repair and apoptosis	*H. sapiens, M. musculus*	[127,128]
H2BS14	Phosphorylated in response to DNA damage, associated with chromatin condensation and apoptosis	*M. musculus*	[182]
H4 S1	Inhibitory to NuA4-mediated acetylation of H4, participates in NHEJ in yeast	*S. cerevisiae*	[183,184]
Acetylation			
H1K85	Recruits HP1 and impedes HR and NHEJ	*H. sapiens*	[185]
H2A.X K5	Mediates release of phosphorylated H2A.X	*H. sapiens*	[186]
H2BK120	Promotes histone H1 eviction, and 53BP1 accumulation over γH2A.X	*M. musculus, H. sapiens*	[109,187]
H3K9,14,18,23,27	GCN5-mediated acetylation in yeast triggered by HR repair. Changes dynamically during DNA repair	*S. cerevisiae*	[188]
H3K14	Globally increased in HMGN-dependent manner in response to IR. Regulates ATM activation	*M. musculus*	[101]
H3K18	Controls the recruitment of Ku proteins	*H. sapiens*	[189]
H3K56	Recruits the chromatin remodeler SNF2H and promotes chromatin relaxation early in the repair process. Forms foci that colocalize with sites of DNA repair Deacetylated rapidly after break induction by HDAC1 and 2, necessary for NHEJ	*M. musculus, Drosophila,* *H. sapiens, S. cerevisiae*	[112,190,191]
H3, H4–N terminus	Acetylated by TRRAP-TIP60 control the recruitment of 53BP1, RAD51, and BRCA1 to DNA damage sites	*H. sapiens*	[192]
H4K5,8,12,16	Controls the recruitment of Ku proteins. GCN5-mediated acetylation in yeast triggered by HR repair. Changes dynamically during DNA repair	*S. cerevisiae, H. sapiens*	[188,189]
H4K16	Deacetylated rapidly after break induction by HDAC1 and -2 promoting NHEJ. Involved in the activation of ATM in response to ionizing radiation	*M. musculus, H. sapiens*	[101,193]
Ubiquitylation			
H1	Serves as a recruitment signal for RNF168, needed for interaction with downstream effectors	*H. sapiens, M. musculus, Gallus gallus*	[58,59,60,132]
H2AK13-15	Critically important for 53BP1 and BRCA1 recruitment	*H. sapiens, M. musculus, Gallus gallus*	[59,60,129,132,194]
H2AK119	Necessary to silence transcription in the vicinity of DSBs and promote HR repair	*H. sapiens, G. gallus*	[116,195,196]
H2BK120	Essential for the timely accumulation of NHEJ and HR proteins at DSB sites (53BP1 and BRCA1 foci formation; XRCC4 and Ku80 recruitment); mediates chromatin relaxation	*H. sapiens, M. musculus*	[107,109,111]
H2AK125, 127, 129	BRCA1/BARD1-mediated ubiquitination. Required for SMARCAD1 binding; promotes DSB end resection.	*S. cerevisae, H. sapiens*	[168,169,170,172]
H4K91	Necessary for H4K20 methylation and 53BP1 foci formation at sites of DNA damage	*H. sapiens*	[197]
H4K119	Induced by H2A.XK5Ac, necessary for the release of phosphorylated H2A.X.	*H. sapiens*	[186]
Methylation			
H3K4me0	Required for ZMYND8-NuRD binding to lesions. NuRD promotes HR repair	*H. sapiens*	[198]
H3K4me2	Reduced at DNA damage sites by LSD1 to promote 53BP1 and BRCA1 foci formation	*H. sapiens, M. musculus*	[199]
H3K9me3	Necessary for binding of HP1β, which is released early during DDR. Directs interaction with TIP60 and controls the recruitment and activity of ATM. Targeted by KDM4D to promote DSB repair	*M. musculus, H. sapiens*	[98,99,200,201]
H3K27me3	Carried out by the EZH2 methyltransferase subunit of PRC2. Required for transcriptional repression around DSBs	*H. sapiens*	[117,202,203]
H3K36me	Promotes chromatin binding of NHEJ factors	*H. sapiens*	[204]
H3K36me2	Increases MRN binding around DSB. Promotes BARD1 binding. Enhances the recruitment of Ku70.	*H. sapiens*	[125,157,204]
H3 K36me3	Stimulates HR repair. Promotes resection via LEDGF-mediated CtIP recruitment	*H. sapiens*	[187,205,206,207,208]
H3 K79me	Promotes binding of 53BP1	*S. cerevisiae, H. sapiens*	[134,149,150,151]
H4 K20me	Necessary for the accumulation of 53BP1 at DSBs.	*S. pombe, M. musculus,* *H. sapiens*	[135,136,137]
H4 K20me2	Required for the binding of L3MBTL1 and TIP60 complex, which compete with 53BP1 for binding sites at DSBs. Necessary for 53BP1 binding	*H. sapiens*	[143,144,147,163,164]

**Table 2:** Chromatin remodelers involved in DSB repair.

Chromatin Remodeler	Function	Model Organism	References
SWI/SNF-family	BRG1 stimulates H2A.X phosphorylation. Binds H2AX-containing nucleosomes via acetylated H3.	*H. sapiens*	[209]
	BRM1 subunit is required for the recruitment of Ku70 and Ku80. BRM1 needs acetylation of H3 by CBP and p300 for its recruitment.	*H. sapiens*	[189]
	BAF complex controls the accumulation of Ku70	*H. sapiens*	[210]
	Human SWI/SNF complex participates in V(D)J recombination.	*H. sapiens*	[211,212,213]
	Yeast SWI/SNF complex participates in HR repair by remodeling nucleosomes at the donor locus. Disrupts heterochromatin by evicting Sir3 from a heterochromatic donor in silent mating-type loci in yeast to facilitate HR repair. Absence of functional SWI/SNF impaired recruitment of MRX and significantly delayed the initiation of DNA end resection.	*H. sapiens,* *S. cerevisiae*	[214,215,216]
	RSC complex is required following synapsis in recombination repair	*H. sapiens*	[214]
	SMARCAD1 is required for the repositioning of 53BP1 away from BRCA1 stimulating HR repair. SMARCAD1 and Fun30 are targeted by CDK1 and together with TopBP1 and yeast Dpb11 facilitate cell cycle-dependent DNA end resection.	*H. sapiens, S. cerevisiae*	[172,174,176,177,178]
	Fun 30 is associated with resection in yeast; influences the distribution of H2A.Z genome-wide and particularly in centromeric, pericentromeric, and subtelomeric chromatin.	*S. cerevisiae,* *H. sapiens*	[174,175,176,217,218,219]
INO80 family	Deficiency of the INO80 complex leads to hypersensitivity to DSB-inducing agents in yeast and HR defects in mammalian cells.	*S. cerevisiae,* *H. sapiens*	[220,221,222]
	INO80 is necessary for the initial 5′- resection at DSB ends prior to strand invasion in both yeast and mammals.	*H. sapiens, S. pombe*, *S. cerevisiae*	[222,223]
	In yeast INO80 mutants loading of Rad51 and Rad52 repair proteins was defective; In higher eukaryotes, INO80 depletion reduces Rad54B and XRCC3 transcription	*S. cerevisiae,* *H. sapiens*	[224,225]
	INO80 complex participates in the maintenance of H2AX phosphorylation levels by antagonizing the SWR1 remodeler.	*S. cerevisiae*	[226]
	Stimulate Rad51 binding to resected DNA during HR repair.	*S. cerevisiae*	[227]
	INO80 promotes nucleosome disassembly (manifested by removal of histone H3 from the genome) during NHEJ	*H. sapiens*	[228]
	Participate in chromatin-bound RNAPIIs degradation in yeast	*S. cerevisiae*	[229]
	Low levels of SRCAP impair resection due to defective CtIP recruitment	*H. sapiens*	[230]
	SWR1 complex maintains H2AX levels by replacing it with H2AZ. Involved in NHEJ, facilitates the recruitment of Ku proteins.	*S. cerevisiae*	[226,231]
	p400 complex deposits H2AZ required for the loading of Ku70/Ku80	*H. sapiens*	[232]
CHD family	CHD1 is required for the recruitment of CtIP and HR repair	*H. sapiens*	[233]
	CHD1B (ALC1) localizes to DSBs in PAR-dependent manner and interacts with Ku70, XRCC1, and DNA-PKcs.	*H. sapiens*	[234]
	CHD2 is involved in the deposition of histone variant H3.3 at sites of DNA damage and efficient assembly of NHEJ complexes	*H. sapiens*	[235]
	CHD4, together with RNF8, creates a chromatin environment that is permissive to the assembly of checkpoint and repair machinery at DSBs	*H. sapiens*	[133,236]
	NuRD complex is implicated in promoting HR repair by repressing transcription at DSBs Recruited to DSBs in a poly(ADP-ribose)-dependent manner, stimulates recruitment of RNF168 and BRCA1 to DSBs. Interacts with Ku70 and is required for the recruitment of Ku proteins at DSBs	*H. sapiens, C. elegans* ** *H. sapiens* ** *H. sapiens, S. pombe*	[237,238,239,240,241]
ISWI family	SNF2H promoteschromatin relaxation early in the repair process; SNF2H is recruited to RNF20-ubiquitylated H2B and its depletion impairs DNA end processing and recruitment of RAD51 and BRCA1.	*H. sapiens*	[110,112]
